# A ketogenic diet impacts markers of mitochondrial mass in a tissue specific manner in aged mice

**DOI:** 10.18632/aging.202834

**Published:** 2021-03-18

**Authors:** Zeyu Zhou, Kevork Hagopian, José A. López-Domínguez, Kyoungmi Kim, Mittal Jasoliya, Megan N. Roberts, Gino A. Cortopassi, Megan R. Showalter, Bryan S. Roberts, José A. González-Reyes, Keith Baar, Jennifer Rutkowsky, Jon J. Ramsey

**Affiliations:** 1 Department of Molecular Biosciences, School of Veterinary Medicine, University of California, Davis, CA 95616, USA; 2 Department of Public Health Sciences, School of Medicine, University of California, Davis, CA 95617, USA; 3 NIH-West Coast Metabolomics Center, University of California, Davis, CA 95616, USA; 4 Department of Cell Biology, Physiology and Immunology, Campus de Excelencia Internacional Agroalimentario, ceiA3, University of Córdoba, Córdoba, Spain; 5 Department of Neurobiology, Physiology, and Behavior, University of California, Davis, CA 95616, USA

**Keywords:** diet, skeletal muscle, brain, liver, kidney

## Abstract

Declines in mitochondrial mass are thought to be a hallmark of mammalian aging, and a ketogenic diet (KD) may prevent the age-related decreases in mitochondrial content. The objective of this study was to investigate the impact of a KD on markers of mitochondrial mass. Mice were fed an isocaloric control diet (CD) or KD from 12 months of age. Tissues were collected after 1 month and 14 months of intervention, and a panel of commonly used markers of mitochondrial mass (mitochondrial enzyme activities and levels, mitochondrial to nuclear DNA ratio, and cardiolipin content) were measured. Our results showed that a KD stimulated activities of marker mitochondrial enzymes including citrate synthase, Complex I, and Complex IV in hindlimb muscle in aged mice. KD also increased the activity of citrate synthase and prevented an age-related decrease in Complex IV activity in aged brain. No other markers were increased in these tissues. Furthermore, the impacts of a KD on liver and kidney were mixed with no pattern indicative of a change in mitochondrial mass. In conclusion, results of the present study suggest that a KD induces tissue-specific changes in mitochondrial enzyme activities, or structure, rather than global changes in mitochondrial mass across tissues.

## INTRODUCTION

Decreases in mitochondrial mass or content are observed in aged animals, and are thought to be a contributor to mammalian aging [1–3]. A ketogenic diet (KD), which is depleted in carbohydrate and high in fat, has been used to manage a range of metabolic and neurologic disorders that are associated with changes in mitochondrial mass, and an upregulation in mitochondrial bioenergetic genes and biogenesis has been proposed as one possible mechanism for the therapeutic and health-promoting effects of this diet [4, 5]. The main circulating ketone body, β-hydroxybutyrate, is also involved in signaling pathways that may lead to an induction in mitochondrial biogenesis [6]. Furthermore, previous studies have demonstrated that a KD increases lifespan and improves physiological functions in aged mice when the intervention was started from middle age [7, 8]. Thus, an increase in mitochondrial mass might be one possible mechanism for the health-promoting effects of this diet. Although a few studies have provided evidence that a KD may increase mitochondrial content in tissues from some animal models [9–11], little is known about the effects of KD on mitochondrial mass in healthy middle-aged and aged mice. The goal of the present study was to determine the impact of a KD on markers of mitochondrial mass in tissues from these animals.

Several markers have been used as measures of mitochondrial mass or content in tissues, with some of the markers showing strong correlation with morphological quantification of mitochondrial content or volume using transmission electron microscopy [12]. The present study focused on the impacts of a KD on a panel of commonly used mitochondrial markers of mitochondrial mass. These markers were selected to allow measurements in whole tissue homogenates and provide an indication of changes in mitochondrial mass across the entire tissue. The markers selected for our study and can be divided into four categories: 1) mitochondrial enzyme activities (citrate synthase, complex I, complex IV); 2) mitochondrial protein levels (citrate synthase, complex I protein NDUFB8, complex IV protein MTCO1); 3) mitochondrial to nuclear DNA ratio; 4) cardiolipin content. We decided to use a broad range of markers to determine if a KD alters tissue mitochondrial mass since each marker has limitations and reliance on a single, or few, markers may give an incomplete picture of mitochondrial changes induced by diet. For example, a change in enzyme activity may reflect post-translational regulatory processes rather than an alteration in mitochondrial content and changes in the levels of a subset of mitochondrial proteins may reflect up or downregulation of a particular pathway rather than an overall change in mitochondrial mass. An increase in all, or the vast majority, of the markers would provide a strong indication of a KD-induced increase in mitochondrial mass. However, changes in only a few of the markers would provide evidence of a KD-related alteration in mitochondrial enzyme activity, or structure, rather than an overt change in mitochondrial mass. To assess the impact of a ketogenic diet on mitochondrial mass a panel of mitochondrial content markers were measured in four metabolically active tissues from C57BL/6JN mice that were fed a control (CD) or ketogenic (KD) diet from 12 months of age (middle age) to 13 or 26 months of age.

## RESULTS

### Ketogenic diet increased activities of mitochondrial enzymes at 26 months of age in hindlimb skeletal muscle without changes in other markers of mitochondrial mass at advanced age

**Figure 1:**
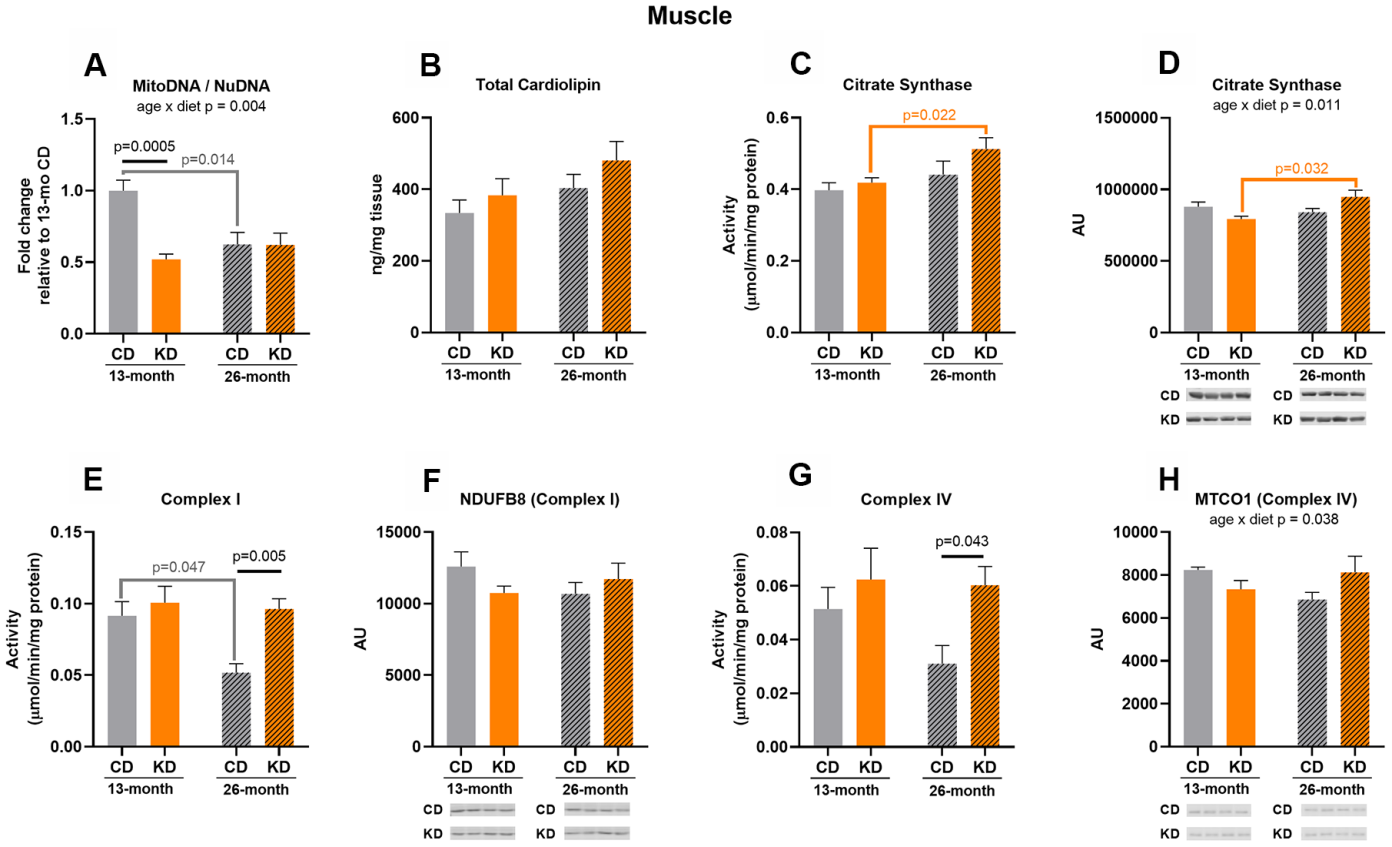
**Markers of mitochondrial content in hindlimb skeletal muscle of male mice after 1 month (13 months of age) and 14 months (26 months of age) on diet (n=4-6).** (**A**) Mitochondrial to nuclear DNA ratio. Quantification of (**B**) total cardiolipin. Enzymatic activities for (**C**) citrate synthase, (**E**) Complex I, and (**G**) Complex IV. Quantification of (**D**) citrate synthase, (**F**) NDUFB8, and (**H**) MTCO1 protein levels by western blots. Diets: CD = control, KD= Ketogenic. Ages: solid bar = 13 months, dashed bar = 26 months. Values are expressed as mean ± SEM. 2-way ANOVAs followed by Bonferroni post hoc tests.

After 1 month of KD (13 months of age), mtDNA to nuDNA ratio was decreased (p = 0.0005) in whole hindlimb skeletal muscle (Figure 1A). No other mitochondrial markers differed between diet groups at 13 months of age (Figure 1B–1H).

After 14 months of intervention (26 months of age), KD mice showed a 2-fold increase in Complex I (p = 0.005) and IV (p = 0.043) activities compared to control mice (Figure 1E–1G). These changes in enzyme activities were not accompanied by elevated enzyme protein levels (Figure 1F–1H) indicating that the increased activities of these enzymes were likely driven by post-translational mechanisms. There were no diet-related changes in other markers of mitochondrial content (Figure 1A–1D). Thus, the changes observed in this age group were only increases in mitochondrial enzyme activities.

Complex I activity in control mice decreased by half (p = 0.047) at 26 months (Figure 1E) while the aged KD mice maintained the activity of this enzyme compared to their younger cohort, and this was not due to age-related changes in NDUFB8 (Complex I) protein level (Figure 1F). There were significant age x diet interactions for mtDNA to nuDNA ratio (Figure 1A), citrate synthase protein level (Figure 1D), and MTCO1 (Complex IV) protein level (Figure 1H) indicating that the diet groups showed a different response to aging. These interactions were accompanied by an age-related decrease in mtDNA to nuDNA ratio (p = 0.014) in the control group (Figure 1A) and an age-related increase in citrate synthase protein (p = 0.032) in the KD mice (Figure 1D). Along with this increase in citrate synthase protein level, citrate synthase activity was increased (p = 0.022) in 26-month old compared to 13-month old KD mice (Figure 1C), and no significant change was observed in old control mice. These results indicate that at the older age KD mice maintained or increased several markers of mitochondrial mass while decreases in some markers were observed in the old control mice. However, neither the KD nor control mice showed a concerted age-related change in the majority of the markers suggesting that there was not an overt change in mitochondrial mass.

### Long-term consumption of a ketogenic diet prevented significant age-related decreases in complex IV activity and stimulated citrate synthase activity in brain but induced few changes in other markers of mitochondrial mass

**Figure 2:**
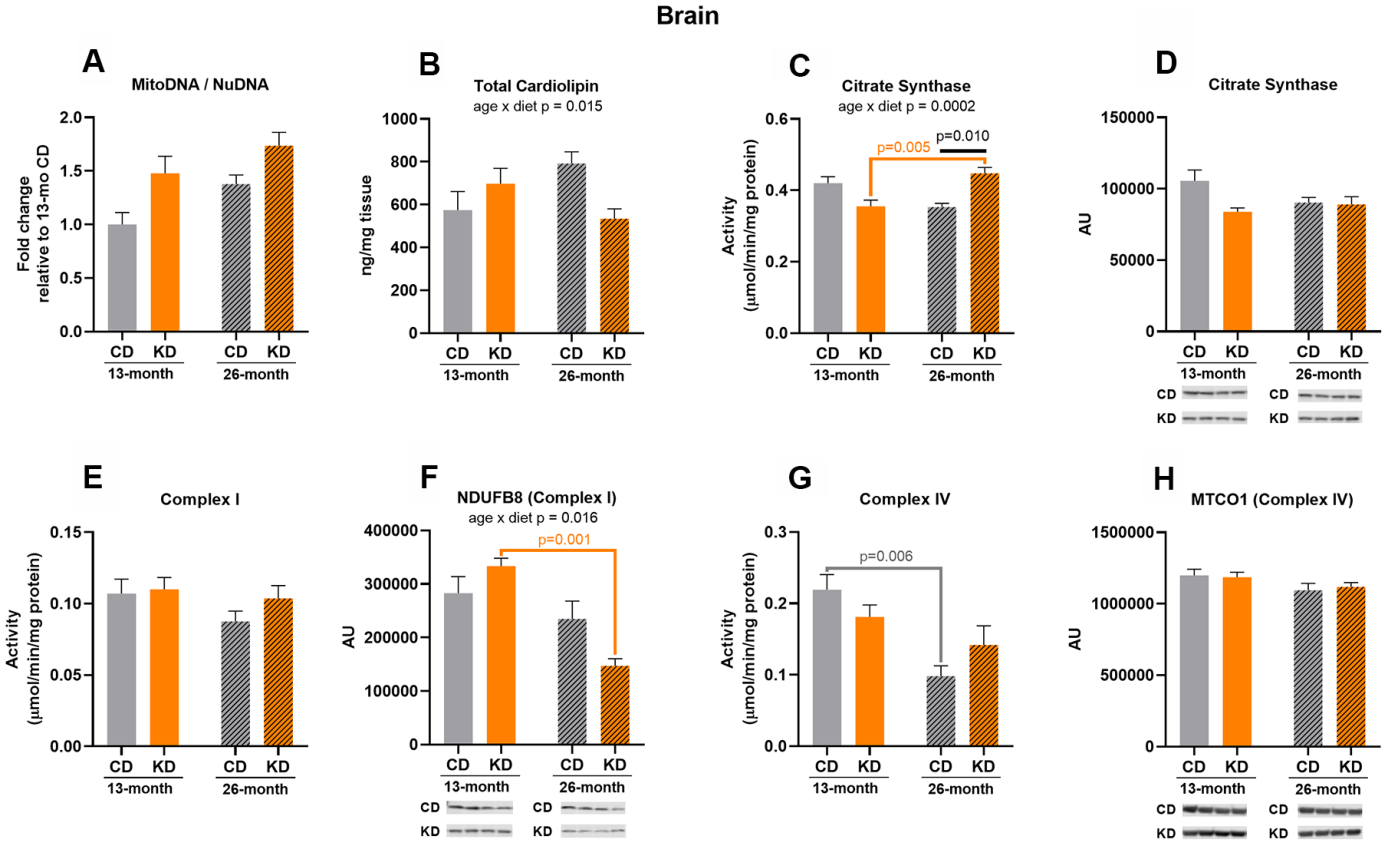
**Markers of mitochondrial content in brain of male mice after 1 month (13 months of age) and 14 months (26 months of age) on diet (n=4-6).** (**A**) Mitochondrial to nuclear DNA ratio. Quantification of (**B**) total cardiolipin. Enzymatic activities for (**C**) citrate synthase, (**E**) Complex I, and (**G**) Complex IV. Quantification of (**D**) citrate synthase, (**F**) NDUFB8, and (**H**) MTCO1 protein levels by western blots. Diets: CD = control, KD= Ketogenic. Ages: solid bar = 13 months, dashed bar = 26 months. Values are expressed as mean ± SEM. 2-way ANOVAs followed by Bonferroni post hoc tests.

After 1 month of KD (13 months of age), there was no significant difference in markers of mitochondrial mass measured between KD and control groups (Figure 2A–2H).

After 14 months of KD (26 months of age), KD mice showed an increase (p = 0.010) in citrate synthase activity compared to the control mice (Figure 2C) and the increase was not caused by changes in protein level (Figure 2D). No other mitochondrial markers differed between diet groups at 26 months of age (Figure 2A, 2B, 2E–2H).

Complex IV activity in control mice decreased by half (p = 0.006) with aging (Figure 2G), while KD mice showed no significant change in the activity of this enzyme with aging. There were also significant age x diet interactions for citrate synthase activity (Figure 2C), NDUFB8 (Complex I) protein (Figure 2F), and cardiolipin (Figure 2B). The interaction for citrate synthase activity was driven by the observation that citrate synthase activity increased (p = 0.005) with aging in the KD, but not control, mice (Figure 2C). In contrast, the interaction for NDUFB8 (Complex I) was due to a decrease by more than half (p = 0.001) in NDUFB8 (Complex I) protein level with aging in the KD, but not control, animals (Figure 2F). It should be noted, however, that this change in protein level was not sufficient to alter Complex I enzyme activity in the KD mice (Figure 2E). There were no other age-related changes in markers of mitochondrial mass (Figure 2A, 2D, 2E, 2H). Some enzyme activities were maintained or increased with aging in the KD mice but there was no change in mitochondrial markers indicative of an increase in brain mitochondrial mass in these animals.

### The effects of ketogenic diet on markers of mitochondrial mass in liver were mixed, indicating no clear pattern of change in mitochondrial mass

**Figure 3:**
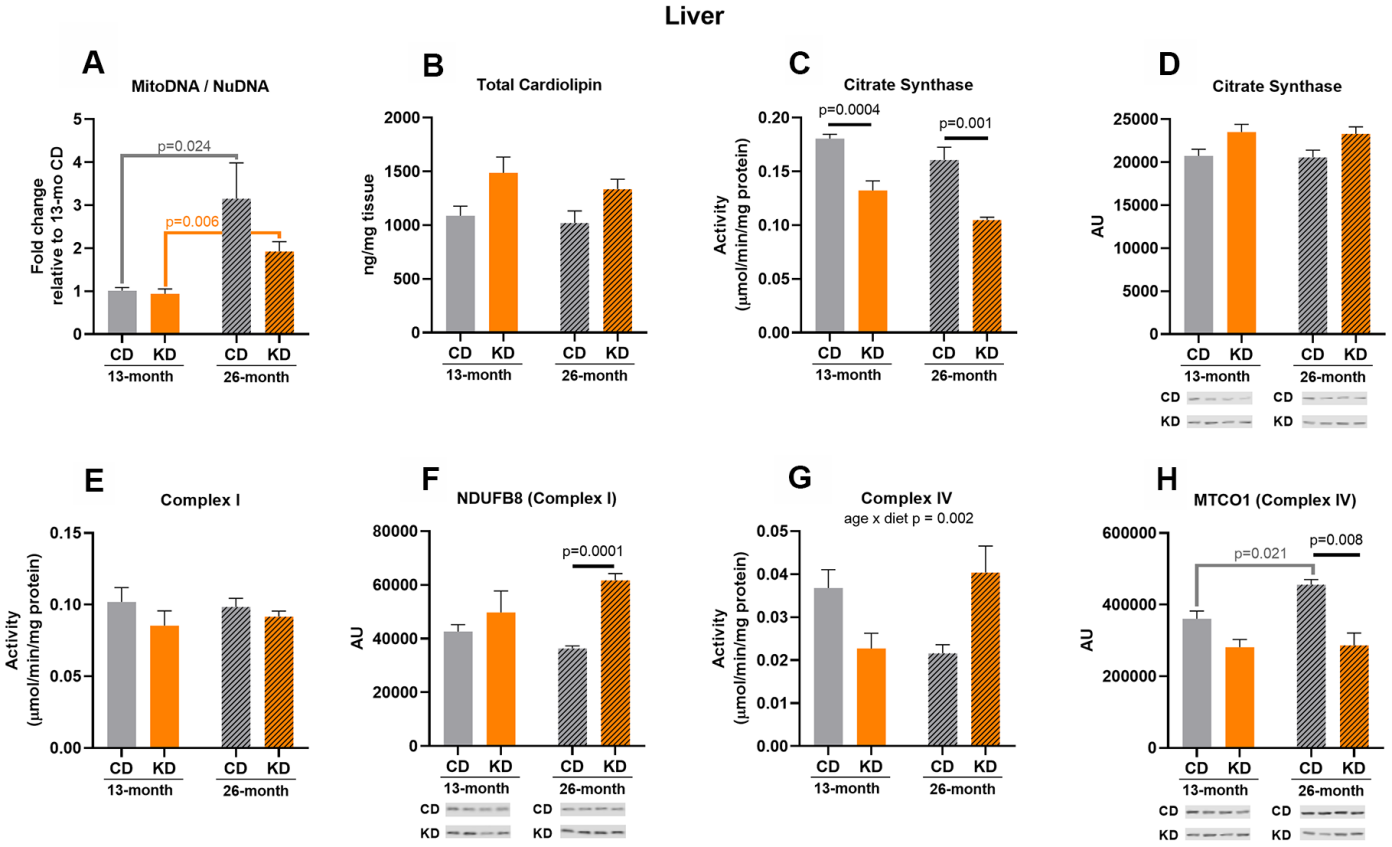
**Markers of mitochondrial content in liver of male mice after 1 month (13 months of age) and 14 months (26 months of age) on diet (n=4-6).** (**A**) Mitochondrial to nuclear DNA ratio. Quantification of (**B**) total cardiolipin. Enzymatic activities for (**C**) citrate synthase, (**E**) Complex I, and (**G**) Complex IV. Quantification of (**D**) citrate synthase, (**F**) NDUFB8, and (**H**) MTCO1 protein levels by western blots. Diets: CD = control, KD= Ketogenic. Ages: solid bar = 13 months, dashed bar = 26 months. Values are expressed as mean ± SEM. 2-way ANOVAs followed by Bonferroni post hoc tests.

Consistent with a chronically activated gluconeogenic pathway in liver of mice on a continuous KD, citrate synthase activity was decreased (p = 0.0004) after 1 month of KD (13 months of age, Figure 3C) and this was not accompanied with a decrease in citrate synthase protein level (Figure 3D). Other markers showed no significant changes with 1 month of KD (Figure 3A, 3B, 3E–3H).

After 14 months of KD (26 months of age), citrate synthase activity was decreased (p = 0.001) compared to control mice (Figure 3C), consistent with the results observed with short-term consumption of a KD. No significant differences were observed in activities of Complex I and IV (Figure 3E–3G), but Complex I (NDUFB8) protein level increased (p = 0.0001) and Complex IV (MTCO1) protein level decreased (p =0.008) in the KD versus control groups (Figure 3F–3H). For the remaining mitochondrial markers, no significant changes were observed (Figure 3A, 3B, 3D).

A significant age x diet interaction was observed in Complex IV activity (Figure 3G) with the KD mice showing a trend (p = 0.056) toward increased Complex IV activity versus decreased (a trend, p = 0.1) activity with aging in the control group. This trend toward an increase in Complex IV activity in the KD mice occurred without age-related changes in MTCO1 (Complex IV) protein levels (Figure 3H). In contrast, MTCO1 (Complex IV) protein level was increased (p = 0.021) with aging in the control animals (Figure 3H). Control and KD mice also showed a 3-fold (p = 0.024) and 2-fold increase (p = 0.006), respectively, in mtDNA to nuDNA ratio with aging (Figure 3A). Citrate synthase activity exhibited a trend (p = 0.053) toward a decrease in the KD mice with aging (Figure 3C). There were no age-related changes in the other markers of mitochondrial mass (Figure 3B, 3D–3F). There was no overall pattern indicative of a change in liver mitochondrial content in aged KD mice or following short or long-term consumption of a KD.

### Mitochondrial markers in kidneys did not show concerted changes indicative of an alteration in mitochondrial mass with a ketogenic diet

**Figure 4:**
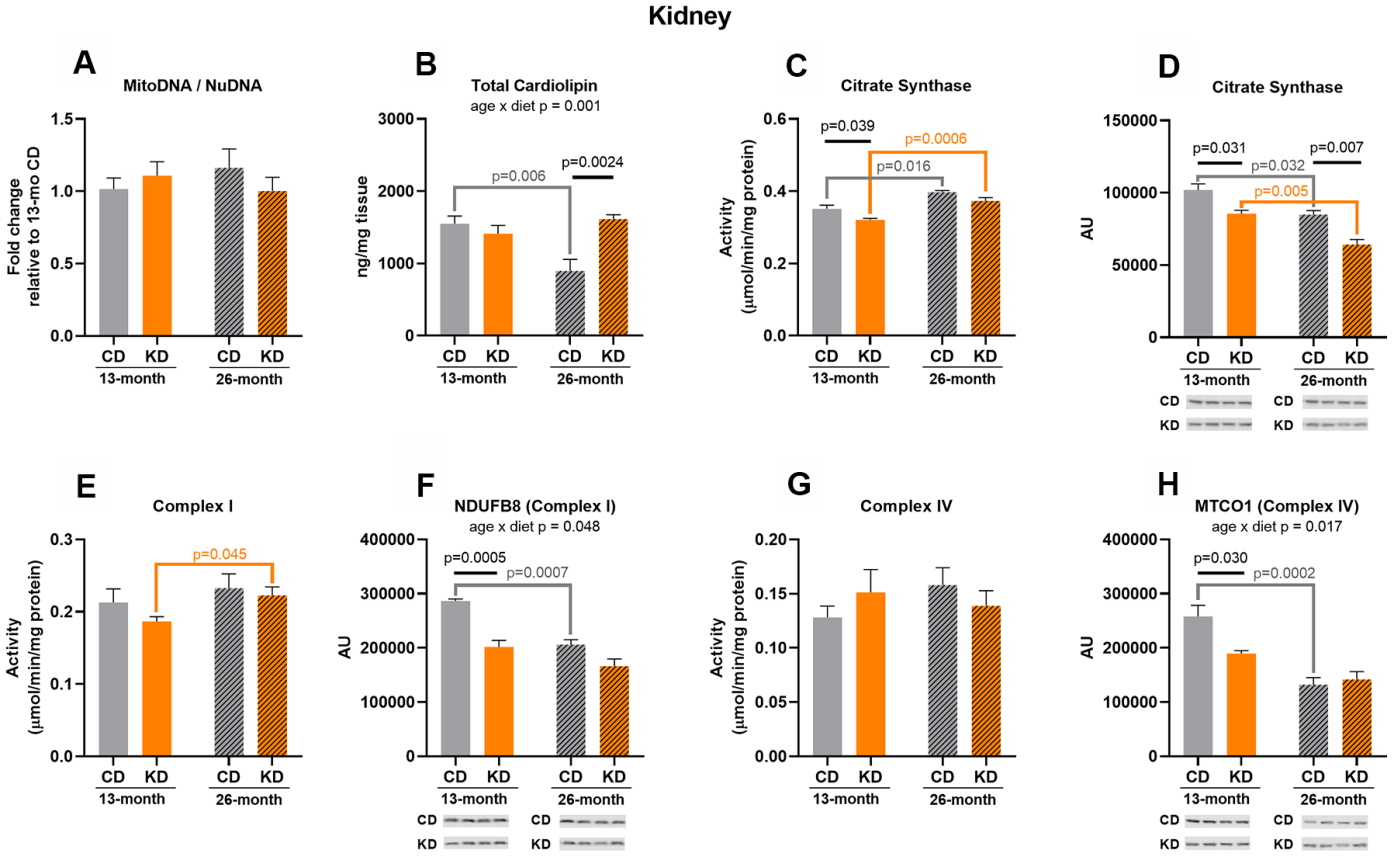
**Markers of mitochondrial content in kidney of male mice after 1 month (13 months of age) and 14 months (26 months of age) on diet (n=4-6).** (**A**) Mitochondrial to nuclear DNA ratio. Quantification of (**B**) total cardiolipin. Enzymatic activities for (**C**) citrate synthase, (**E**) Complex I, and (**G**) Complex IV. Quantification of (**D**) citrate synthase, (**F**) NDUFB8, and (**H**) MTCO1 protein levels by western blots. Diets: CD = control, KD= Ketogenic. Ages: solid bar = 13 months, dashed bar = 26 months. Values are expressed as mean ± SEM. 2-way ANOVAs followed by Bonferroni post hoc tests.

Citrate synthase activity was decreased (p = 0.039) in KD mice after 1 month on diet (13 months of age, Figure 4C), and this may in part be due to a decrease (p = 0.031) in citrate synthase protein level (Figure 4D). After 1 month of KD, Complex I (NDUFB8) and IV (MTCO1) protein levels were lower (p =0.0005, p = 0.030) in the KD versus control groups (Figure 4F–4H) but the activities of these enzymes were not significantly different (Figure 4E–4G) between diet groups. There were no changes in other markers between diet groups at 13 months of age (Figure 4A, 4B).

In the 26-month old group (14 months on diet), cardiolipin content was 2-fold higher (p = 0.0024) in old KD versus control mice (Figure 4B) implying an increase in inner membrane lipid amount. KD mice showed a decrease (p = 0.007) in citrate synthase protein level (Figure 4D) consistent with the results observed in the 13-month old group, but this did not translate to a significant decrease in citrate synthase activity (Figure 4C). There were no changes in other markers between diet groups at 26 months of age (Figure 4A, 4E–4H).

There were significant age x diet interactions for cardiolipin (Figure 4B), NDUFB8 (Complex I) (Figure 4F), and MTCO1 (Complex IV) (Figure 4H). All of these interactions were driven by a significant (p < 0.05) age-related decrease in the control, but not KD, mice. In contrast to liver, there was an age-related increase in citrate synthase activity in both the control (p = 0.016) and KD (p = 0.0006) groups (Figure 4C), but this was accompanied by a decrease (p= 0.032 and 0.005, respectively) in citrate synthase protein abundance with aging in both diet groups (Figure 4D). Complex I activity was also increased (p = 0.045) with aging in the KD group (Figure 4E) and this was not caused by a change in protein level (Figure 4F). There were no age-related changes in mtDNA to nuDNA ratio (Figure 4A) or Complex IV activity (Figure 4G). Similar to liver, there was no overall pattern indicative of a change in kidney mitochondrial mass in aged KD mice or following short or long-term consumption of a ketogenic diet.

## DISCUSSION

The effects of KDs on measures of mitochondrial mass have been reported in laboratory rodents. However, these studies have almost exclusively focused on models of injury or disease [11, 13–15] or used only one marker of mitochondrial mass collected from moribund animals [10]. Thus, relatively little is known about the impact of long-term consumption of a KD on markers of mitochondrial mass and it is not clear if a KD increases mitochondrial mass in particular tissues.

This study is the first to report the influence of a KD on a panel of markers of mitochondrial mass in various tissues of healthy middle-aged (13-month) and aged (26-month) mice.

Although there is evidence that KDs increase transcription of proteins involved in mitochondrial energy metabolism [9], increase markers of mitochondrial biogenesis [16], and induce post-translational modification of mitochondrial proteins [8, 17], there is currently no convincing evidence showing an increase in mitochondrial content across multiple tissues with a KD. The results of the present study did not provide evidence of a widespread increase in mitochondrial mass. We did not observe concerted changes in all markers of mitochondrial mass in any tissue implying the intervention may be inducing remodeling of the organelle or impacting only some mitochondrial components rather than inducing a broad increase in mitochondrial mass. This conclusion would be consistent with the fact that animals on a KD did not have increased whole animal energy expenditure [7]. Unlike exercise training, where a clear increase in mitochondrial mass has been observed in skeletal muscle [18], there is not a similar drive to increase capacity for mitochondrial energy production in animals consuming a KD. The need to change fuel utilization, rather than increase energy production, might contribute to the changes observed in the present study where the KD differentially altered markers of mitochondrial content in a tissue-specific manner.

The impact of a KD on mitochondrial mass in skeletal muscle is of particular interest since this post-mitotic tissue is rich in mitochondria and shows an age-related decline in function. Among the markers of mitochondrial mass, activities of ETC enzymes and citrate synthase have shown a strong association with skeletal muscle mitochondrial mass [3]. Our results demonstrate that the activities of Complexes I and IV were increased in hindlimb skeletal muscle after 14 months of a KD and these are consistent with the improved muscle strength observed in our previous study [7]. Little is known about the impact of KDs on skeletal muscle ETC enzyme activities, with one study reporting no change in ETC enzyme activities in rats consuming a KD for 6 weeks [19]. This finding is consistent with our 1-month results and indicates that duration on diet or age influences the increases in Complex I and IV activities we observed in mice with long-term KD. These increases in enzyme activities on a long-term KD, however, do not appear to be indicative of an increase in mitochondrial content since they were not accompanied by similar changes in Complex I or IV protein levels. Thus, the impact of a KD on these enzymes seems to occur primarily through post-translational mechanisms [20, 21]. Increased level of lysine acetylation and β-hydroxybutyrylation are potential post-translational modifications of mitochondrial enzymes under KD [22], but whether these modifications to citrate synthase, Complex I, or Complex IV impact enzyme activities is not clear [23, 24]. More studies are needed to elucidate the mechanisms underlying the changes of mitochondrial enzyme activities under KD. In regard to the influence of a KD on citrate synthase activity, we did not observe a difference between diet groups. Other KD studies in rodents have shown no consensus with gastrocnemius citrate synthase activity decreased [25], increased [10], or unchanged [11, 19] with a KD. These studies differed in animal age, duration on diet and timing of sample collection in relation to feeding, and there is not sufficient information at this time to determine what factors are driving the differences in citrate synthase activity between these KD studies. The existing citrate synthase data provide little evidence for a clear KD-induced increase in hindlimb skeletal muscle mitochondrial content. In addition to changes in mitochondrial enzymes, the mtDNA to nuDNA ratio has been used as an indicator of mitochondrial mass [26]. We found no evidence for an increase in the mtDNA to nuDNA ratio with the KD. In fact, the KD group actually showed a decrease at 1 month of dietary intervention. An age-related decrease in mtDNA content has been shown in several [27–29], but not all [26] studies using rodent skeletal muscle. In our study, the control mice showed an age-related decrease in mtDNA to nuDNA ratio while no change was observed in KD mice. Similarly, the KD group did not show age-related decreases in Complex I enzyme activity that was observed in the control group. Long-term consumption of a ketogenic diet increased ETC enzyme activities at the older age and prevented age-related changes in some mitochondrial components that occurred in skeletal muscle in old control mice, although all these changes appeared to be through mechanisms distinct from an overall change in mitochondrial mass. Our results suggest that a KD may promote maintenance of hindlimb skeletal muscle mitochondria with age rather than inducing an overall increase in mitochondrial mass across all hindlimb muscle groups.

The influence of a KD on brain mitochondrial mass was also of interest since we previously observed improved memory in aged mice fed a KD [7]. The use of KDs to manage age-related neurological disorders has been actively explored [30] and KDs might provide benefits by stimulating cellular energy metabolism and mitochondrial biogenesis in brain [31]. However, relatively little is known about the impact of sustained consumption of a KD on markers of mitochondrial mass. Activities of citrate synthase and ETC enzymes have been reported to decrease with aging in rodent [32–35] and primate [36] brains and KD-induced changes in mitochondrial content could oppose these age-related changes. However, a study in rats reported that consumption of a KD for 8 months did not have an impact on citrate synthase activity in cortex [25]. The influence of a KD on brain citrate synthase in our study was complex with one month of diet inducing a trend toward decreased activity and protein level, while long-term consumption of a KD resulted in an increase in citrate synthase activity that was not driven by changes in protein amount. Our results suggest that citrate synthase shows a time dependent response to a KD that depends on diet duration and/or age. In regard to ETC enzymes, it was reported that 1 month of a KD did not change the levels of subunits of ETC proteins [37], which is consistent with our findings. Similarly, 14 months of KD did not alter ETC enzyme activities although it did prevent age-related decreases in Complex IV activity observed in the control group. The KD was able to maintain ETC enzyme activities despite a decrease in Complex I (NDUFB8) protein, providing further evidence that a KD may be altering the activities of some proteins through post-translational modifications. Collectively, our results for brain indicate that long-term consumption of a KD maintained Complex IV activity and increased citrate synthase activity with aging, but these changes do not appear to be due to an overall increase in brain mitochondrial content since similar increases in mtDNA to nuDNA ratio, cardiolipin or protein levels were not observed. The disparate changes in markers of brain mitochondrial mass with consumption of a KD suggests only a partial stimulation of mitochondrial pathways rather than a clear increase in mitochondrial mass.

A limitation of our skeletal muscle and brain work was that the mitochondrial markers were analyzed in homogenates of the entire hindlimb skeletal muscle and brain, and it was not possible to determine the response of specific muscles or brain regions to a KD. Variable impacts of aging on markers of mitochondrial content have been found in different rodent hindlimb muscles [29]. Changes in muscle fiber type composition, which can have an impact on mitochondrial mass in muscle, have been reported with aging and calorie restriction [38, 39], and the effect of KD on fiber types should be explored in future studies to elucidate if the changes in markers of mitochondrial mass are correlated to alterations in fiber type composition. In brain, variations in cell architecture and population can occur with aging and region specific changes in brain mitochondrial mass have also been observed [40, 41]. Studies using transmission electron microscopy (TEM) have shown that KDs may increase mitochondrial abundance under certain conditions in some neural populations [9, 41–43]. Measurements of markers of mitochondrial mass or mitochondrial quantification using TEM will be needed for future studies in multiple muscles and brain regions to determine if a KD alters mitochondrial mass in specific muscle groups or regions of the brain.

Liver is involved in many metabolic adaptations to a KD including increased fatty acid oxidation, ketone body production, and gluconeogenesis, and these metabolic alterations might have an impact on some markers of mitochondrial mass. Liver citrate synthase activity was decreased with the KD in our study, which could reflect chronically stimulated gluconeogenesis [44]. This could be explained by the fact that citrate synthase is inhibited by palmitoyl-CoA, a metabolite increased during ketosis [45]. However, the impact of a KD on citrate synthase activity likely depends on diet composition and physiological state. Rat studies have shown that citrate synthase activity increased [10] or stayed unchanged [25] in liver with a KD. However, the study that reported no change had much higher protein and carbohydrate levels than the diet used in our study, limiting the necessity for gluconeogenesis. The animals on this diet also did not have significantly higher β-hydroxybutyrate level compared to control rats. Animals in the study that found an increased activity were moribund when tissues were collected, and this extreme metabolic state makes it hard to compare these results to healthy animals. Another rat study found that a KD combined with voluntary wheel running increased liver citrate synthase activity [16]. Liver samples from these rats were collected following a brief fast (3-4 hours) and insulin levels were above typical fasting levels suggesting that gluconeogenesis may have been inhibited at the time of sample collection in this study. Thus, citrate synthase activity is influenced by the metabolic need of the animal, leading to variability in results observed between studies and making the activity of this enzyme a poor marker for liver mitochondrial content. Moreover, a study reported that a KD differentially altered mitochondrial matrix and inner membrane protein levels in 5-month old mice [17], which are consistent with the mixed changes in citrate synthase, Complex I, and Complex IV protein levels we observed with the consumption of a KD in our study. Previous studies have demonstrated an increase [46, 47] or decrease [48, 49] in mtDNA content in aged rodents, and in this study a significant increase in mtDNA to nuDNA ratio with aging was observed for both control and KD mice, with no diet-related difference. The increase in mtDNA to nuDNA ratio was not likely due to an increase in overall mitochondrial mass as most of the other markers were not changed with aging. Although some significant changes in makers of mitochondrial mass were observed, the lack of a common direction in these changes did not provide support for an alteration in mitochondrial mass between KD and control groups. Overall, liver did not show coordinated changes in mitochondrial markers consistent with an increase in mitochondrial mass, and instead, the mitochondrial changes likely reflected the sustained shift in metabolic pathways induced by long-term consumption of a KD rather than a change in capacity for energy metabolism.

Little is known about the influence of KD and aging on markers of mitochondrial mass in kidneys. Abnormalities in mitochondrial morphology have been observed in kidneys of aged rodents [50] and our results demonstrated that aging decreased cardiolipin content, a marker of mitochondrial membrane content, and the KD prevented the age-related declines observed in the control mice. The results in kidneys also suggest that a KD may help maintain mitochondrial components with aging, but this does not appear to be due to an overall change in mitochondrial mass.

Our data demonstrated that there is no uniform change with a KD in the markers of mitochondrial mass assayed in this study either within or across tissues. Discrepancies in outcomes of dietary interventions may be the result of drawing conclusions from a single marker of mitochondrial mass and the use of different markers between studies. For example, mtDNA content, as mtDNA copy number or mtDNA to nuDNA ratio, has been commonly used as a major marker of mitochondrial content. However, there are instances when mitochondrial biogenesis is not accompanied with an increase in mtDNA [51], and a study in human skeletal muscle found that mtDNA was a poor marker of mitochondrial content when compared to measurements using TEM [12]. Thus, a single or a few markers may not reflect actual changes in mitochondrial mass, and a variety of markers is likely required to thoroughly determine if an intervention increases mitochondrial mass or differentially affects components of mitochondria to induce remodeling of the organelle. Tissue specific differences were also observed in the study, and more work is needed to determine which markers best reflect changes in mitochondrial mass in each tissue.

In summary, our study demonstrated that a KD affected markers of mitochondrial mass or content in a tissue specific manner in male C57BL/6JN mice. Stimulation of mitochondrial enzymes was observed in skeletal muscle and brain, likely due to post-translational modifications of the enzymes rather than an increase in amount of enzyme or mitochondria. Age-related changes in some markers were also prevented by the KD. The impacts of KD on liver and kidney markers were mixed and likely reflect regulation or remodeling of mitochondria to meet metabolic demands of the animal. The results of the present study suggest that a KD induces tissue-specific changes in mitochondrial enzyme activities, or structure, rather than global changes in mitochondrial mass across tissues. Moreover, measurements using multiple muscle groups and brain regions are needed for future studies to investigate changes of mitochondrial mass in specific tissue groups or regions in response to a KD.

## MATERIALS AND METHODS

### Animals and diets

Male C57BL/6JN mice were obtained at 11 months of age from the NIA Aged Rodent Colony. Mice were individually housed in polycarbonate cages on racks in a HEPA filtered room maintained on a 12-hour light-dark cycle. Temperature (22–24° C) and humidity (40– 60%) were controlled and health checks were conducted on all mice at least once daily. Sentinel mice were housed in the same room and exposed to bedding from the study mice on a weekly basis. Health screens were completed on sentinel mice every three months. Tests included aerobic cultures and serology (MHV, MPV, MVM, M. pul., TMEV [GDVII], Ectro, EDIM, MAD1, MAD2, LCM, Reo-3). All tests were negative throughout the study. All animal protocols were approved by the UC Davis Institutional Animal Care and Use Committee and were in accordance with the NIH guidelines for the Care and Use of Laboratory Animals.

Upon arrival at the UC Davis facility, mice were singly housed and provided ad libitum access to a chow diet (LabDiet 5001; LabDiet, Saint Louis, MO) and food intake was measured. At 12 months of age, mice were randomly assigned to a control (CD) or ketogenic (KD) diet. The control diet contained (% of total kcal) 18% protein, 65% carbohydrate, and 17% fat. The ketogenic diet contained 10% protein, <1% carbohydrate, and 89% fat. The animals were fed 11.2 kcal/day. Table 1 provides a detailed description of the diet composition. For the control diet, the Envigo (Indianapolis, IN) mineral mix TD.94046 and the vitamin mix TD.94047 were used. The KD included mineral mix TD.79055 and vitamin mix TD.40060 instead, because of their lower carbohydrate content. Thus, calcium phosphate (19.3 g/kg diet) and calcium carbonate (8.2 g/kg diet) supplementation was required for the KD. The vitamin mix added to the KD included choline (choline dihydrogen citrate) for a final concentration of 4.5 g/kg of diet. Nutritional ketosis was confirmed by measuring blood ketone levels using a Precision Xtra glucose and ketone monitoring system (Abbott) through tail snips and the data were provided in our previously published paper [7].

**Table 1:** Diet ingredients.

**G/kg diet**	**Control**	**Ketogenic**
Protein, of which	203.0	183.7
Casein	200	181
L-Cysteine	3.0	--
D-methionine	--	2.7
Carbohydrates, of which	630	--
Corn starch	398	--
Maltodextrin	132	--
Sucrose	100	--
Fats, of which	70.0	631.0
Soybean oil	70	70
Lard	0	561
Choline bitartrate	2.5	--
Cellulose	50	85.0
TBHQ	0.014	0.126
Mineral mix	35	60
Vitamin mix	10	13

After 1 or 14 months of dietary intervention, animals were sacrificed with cervical dislocation and tissues were collected in the morning following an overnight fast. Tissues were collected and snap frozen in liquid nitrogen. The entire liver, both kidneys, whole brain (including olfactory bulb and medulla), and all the muscles from the hindlimb were harvested.

### Mitochondrial enzyme activities

Tissues were powdered under liquid nitrogen using a mortar and pestle. The powdered tissues were homogenized at a 1:10 (w/v) tissue to buffer ratio in a glass homogenizer. Citrate synthase, Complex I and Complex VI activity was measured as described previously [52, 53]. Assays were performed in cuvettes with a final volume of 1 mL, using a Perkin Elmer Lambda 25 UV/VIS spectrophotometer (Waltham, MA) equipped with Peltier heating control system and a 9-cell changer. Enzyme activities were expressed as μmol/min/mg protein.

### Total cardiolipin content

Measurements were carried out on an Agilent 6530a Q-TOF instrument (Santa Clara, CA) following lipid extraction from 6 mg of tissue at the West Coast Metabolomics Center (Davis, CA). A calibration curve was run for cardiolipin quantification using CL 72:8 at concentrations 0.001μg/mL-100μg/mL. Details regarding lipid extraction and instrumentation are described in the Supplementary Material.

### mtDNA to nuDNA ratio

Quantitative PCR of a mitochondrial gene mtND1 (mitochondrially encoded NADH dehydrogenase 1) relative to a single copy nuclear gene, Cftr (Cystic fibrosis transmembrane conductance regulator) was used to measure mtDNA to nuDNA ratio. Total DNA was extracted from tissues using a DNeasy blood and tissue kit (Qiagen, Valencia, CA) and quantified using a NanoDrop 2000c Spectrophotometer (Thermo

Scientific, Waltham, MA). A SensiFAST SYBR No-ROX Kit (Bioline, Taunton, MA) was used to perform qPCR in a Roche Lightcycler 480 (Roche Diagnostics, Indianapolis, IN, USA). Primer sequence targeting mtND1 and Cftr includes, mt-Nd1-F: TCCGAGCATCTTATCCACGC, mt-Nd1-R: GTATGGTGGTACTCCCGCTG, Cftr-F:ATGGTCCACAATGAGCCCAG, Cftr-R:GAACGAATGACTCTGCCCCT). The second derivative of the amplification curve was used to determine the cycle threshold, and the data were analyzed by a delta-delta CT calculation.

### Western blotting

Powdered tissue samples were homogenized in a RIPA lysis buffer (Cell Signaling, Danvers, MA,) with a protease inhibitor cocktail (Roche, Basel, Switzerland). Samples were centrifuged at 16,000 g for 20 minutes and supernatant was collected. Protein concentration was measured using a Bradford protein assay kit (Bio-Rad, Hercules, CA, USA) and 30 μg of protein was loaded onto a 4-20% SDS-PAGE gel (Bio-Rad) and run at 200 V for 45 minutes. The protein was transferred to a 0.2 μm nitrocellulose membrane (Bio-Rad) at 100 V for 30 minutes using the plate electrode. Total protein normalization was assayed using a Revert 700 Total Protein Stain Kit (LI-COR, Lincoln, NE, USA) Supplementary Figures 1–4. The membrane was blocked using a blocking buffer (LI-COR) then probed with primary antibody overnight at 4° C. Primary antibodies against MTCO1 (Complex IV) (ab14705,1:2000; ab203912, 1:1000), NDUFB-8 (Complex I) (ab110242, 1:2000), and Citrate Synthase (ab129095, 1:1000) were obtained from Abcam (Cambridge, United Kingdom). NIR fluorescent secondary antibodies (LI-COR) were applied and membranes were imaged using a LI-COR Odyssey imager.

### Statistical analysis

All values are expressed as mean ± SEM unless otherwise indicated. Diet and aging effects on markers of mitochondrial mass were determined using two-way ANOVAs with main effects of diet and age and their interaction, followed by Bonferroni post hoc tests. Significance for all comparisons was set at p < 0.05, while controlling for multiple testing by Bonferroni method. All statistical analyses were conducted using GraphPad Prism 8.1 (GraphPad Software Inc., San Diego, CA).

## Supplementary Material

Supplementary Methods

Supplementary Figures
