# Supplementary material for: A ketogenic diet impacts markers of mitochondrial mass in a tissue specific manner in aged mice
Source: Aging (Albany NY). 2021 Mar 18;13(6):7914–30. doi: 10.18632/aging.202834 (PMC8034930; doi:10.18632/aging.202834)
Supplement: Supplementary Methods [file aging-13-202834-s001.pdf]

SUPPLEMENTARY MATERIALS

Cardiolipin measurement

Lipids were extracted from 6 mg of tissue with methanol and methyl tert-butyl ether. Water was subsequently added for phase separation. After concentrating extracts to complete dryness, samples were reconstituted prior to LC-MS analysis in 110 µL of methanol:toluene (90:10, v/v) with 50 ng/mL CUDA standard.

All measurements were carried out on an Agilent 6530a Q-TOF instrument [1]. For positive mode, 10 µL of diluted samples were injected. For negative mode, 1uL of diluted samples were injected. Samples were separated on a Waters Acquity UPLC CSH C18 column (100 × 2.1 mm; 1.7 µm) coupled to an Acquity UPLC CSH C18 VanGuard precolumn (5 × 2.1 mm; 1.7 µm). The column was maintained at 65° C with a flow rate of 0.6 mL/min. The positive ionization mobile phases consisted of (A) acetonitrile:water (60:40, v/v) with ammonium formate (10 mM) and formic acid (0.1%) and (B) 2-propanol:acetonitrile (90:10, v/v) with ammonium formate (10 mM) and formic acid (0.1%). The negative ionization mobile phases consisted of (A) acetonitrile:water (60:40, v/v) with ammonium formate (10 mM) and (B) 2-propnol:acetonitrile (90:10, v/v) with ammonium formate (10 mM). The separation was conducted under the following gradient: 0 min 15% B; 0−2 min 30% B; 2−2.5 min 48% B; 2.5−11 min 82% B; 11−11.5 min 99% B; 11.5−12 min 99% B; 12−12.1 min 15% B; 12.1−15 min 15% B.

The Agilent 6530a QTOF instrument was operated using positive mode electrospray ionization using the following parameters. Acquisition parameters: Mass range, 120-1700 m/z; Acquisition rate, 2 spectra/second; Acquisition time, 500 ms/spectrum; Mode, MS(Seg). Source Parameters: Gas Temp, 325° C; Drying Gas, 8L/min; Nebulizer, 35psig; Sheath Gas Temp, 350° C; Sheath gas flow, 11L/min; VCap, 3500V; Spectrum data type, Centroid. MS TOF parameters: Fragmentor, 120V; Skimmer, 65V; OCT 1 RP Vpp, 750V, Collision Energy, 0V. MSMS were acquired in a separate injection using the following acquisition parameters: MS1 Mass range, 65-1700m/z; MS/MS mass range, 35-1700; MS1 acquisition rate, 4 spectra/second; MS1 acquisition time, 250 ms/spectrum; MS/MS acquisition rate, 8 sepctra/s; MS/MS acquisition time, 125 ms/spectrum. Collision energy depended on m/z and was calculated using the following formula: 3×((m/z)/100)+2.5. Source parameters for MS/MS injections were the same as MS injections.

The Agilent 6530a QTOF instrument was operated using negative mode electrospray ionization using the following parameters. Acquisition parameters: Mass range, 60-1700 m/z; Acquisition rate, 2 spectra/second; Acquisition time, 500 ms/spectrum; Mode, MS(Seg). Source Parameters: Gas Temp, 325° C; Drying Gas, 8L/min; Nebulizer, 35psig; Sheath Gas Temp, 350° C; Sheath gas flow, 11L/min; VCap, 3500V; Spectrum data type, Centroid. MS TOF parameters: Fragmentor, 120V; Skimmer, 65V; OCT 1 RP Vpp, 750V, Collision Energy, 0V. MSMS were acquired in a separate injection using the following acquisition parameters: MS1 Mass range, 65-1700m/z; MS/MS mass range, 35-1700; MS1 acquisition rate, 4 spectra/second; MS1 acquisition time, 250 ms/spectrum; MS/MS acquisition rate, 8 sepctra/s; MS/MS acquisition time, 125 ms/spectrum. Collision energy depended on m/z and was calculated using the following formula: 3×((m/z)/100)+2.5. Source parameters for MS/MS injections were the same as MS injections.

A calibration curve was run for cardiolipin quantification using CL 72:8. The curve was run at the following concentrations: 0.001µg/mL, 0.01µg/mL, 0.1µg/mL, 1µg/mL, 10µg/mL, 20µg/mL, 50µg/mL, 100µg/mL. The LC-MS/MS data was analyzed by MS-DIAL software.

REFERENCE

1. Tsugawa H, Ikeda K, Takahashi M, Satoh A, Mori Y, Uchino H, Okahashi N, Yamada Y, Tada I, Bonini P, Higashi Y, Okazaki Y, Zhou Z, et al. A lipidome atlas in MS-DIAL 4. Nat Biotechnol. 2020; 38:1159–63. https://doi.org/10.1038/s41587-020-0531-2 PMID: 32541957
