# Supplementary material for: A ketogenic diet impacts markers of mitochondrial mass in a tissue specific manner in aged mice
Source: Aging (Albany NY). 2021 Mar 18;13(6):7914–30. doi: 10.18632/aging.202834 (PMC8034930; doi:10.18632/aging.202834)
Supplement: Supplementary Figures [file aging-13-202834-s002.pdf]

SUPPLEMENTARY FIGURES

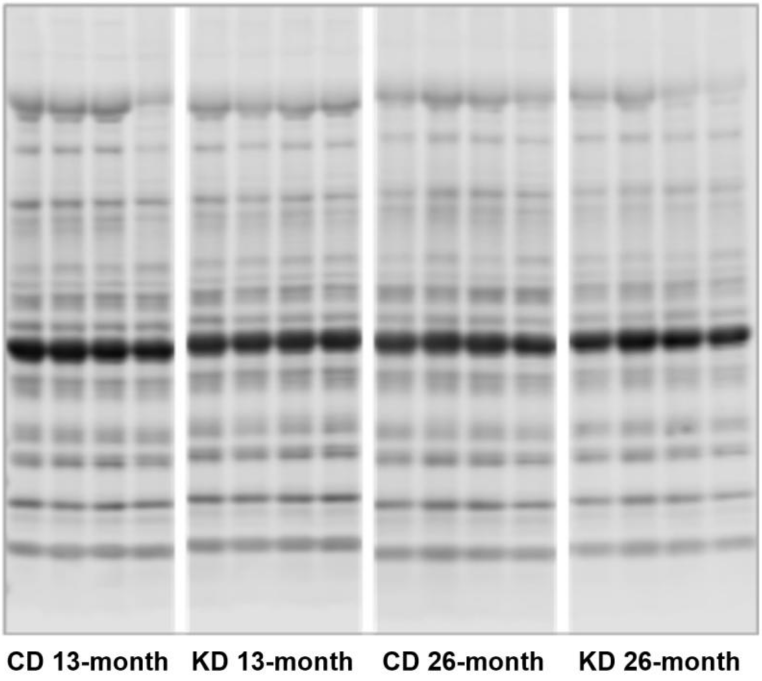

Supplementary Figure 1. Total protein normalization for western blot analysis of hindlimb muscle.

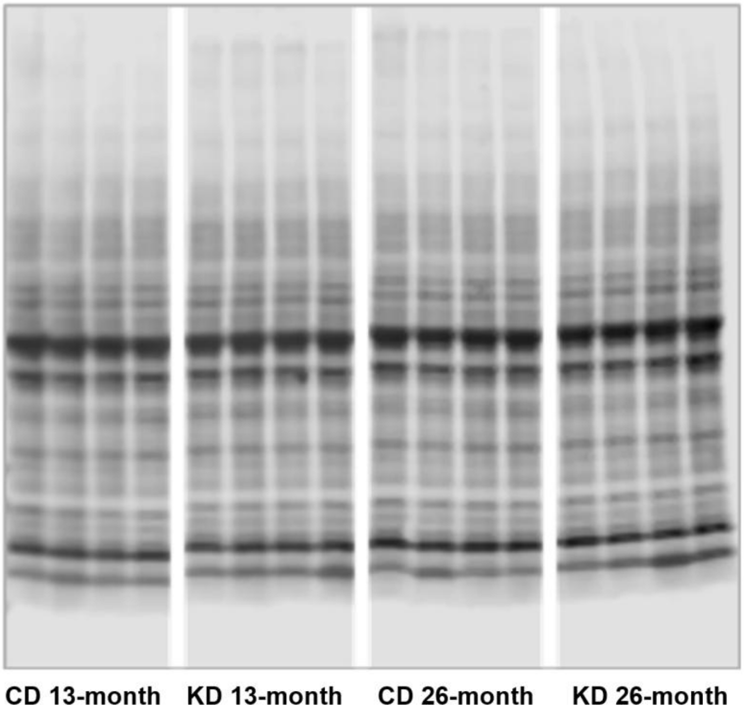

Supplementary Figure 2. Total protein normalization for western blot analysis of brain.

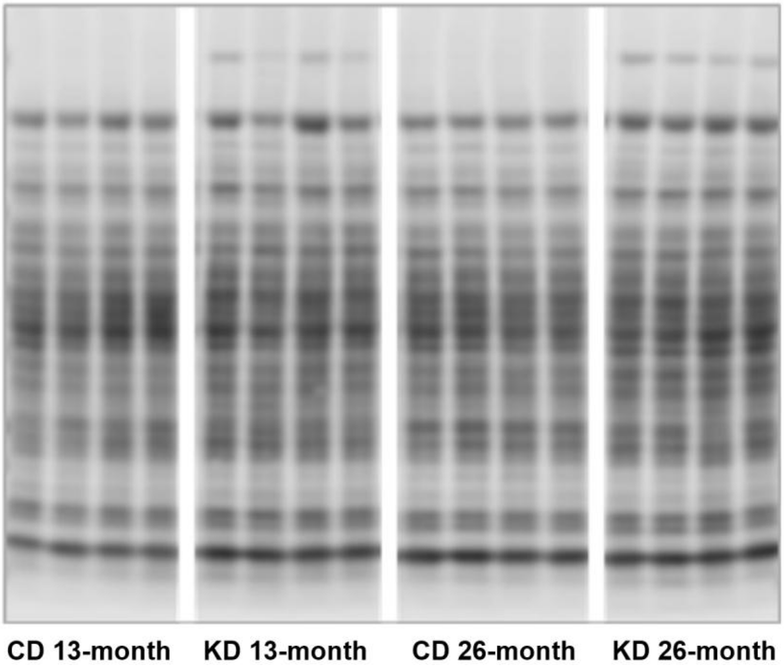

Supplementary Figure 3. Total protein normalization for western blot analysis of liver.

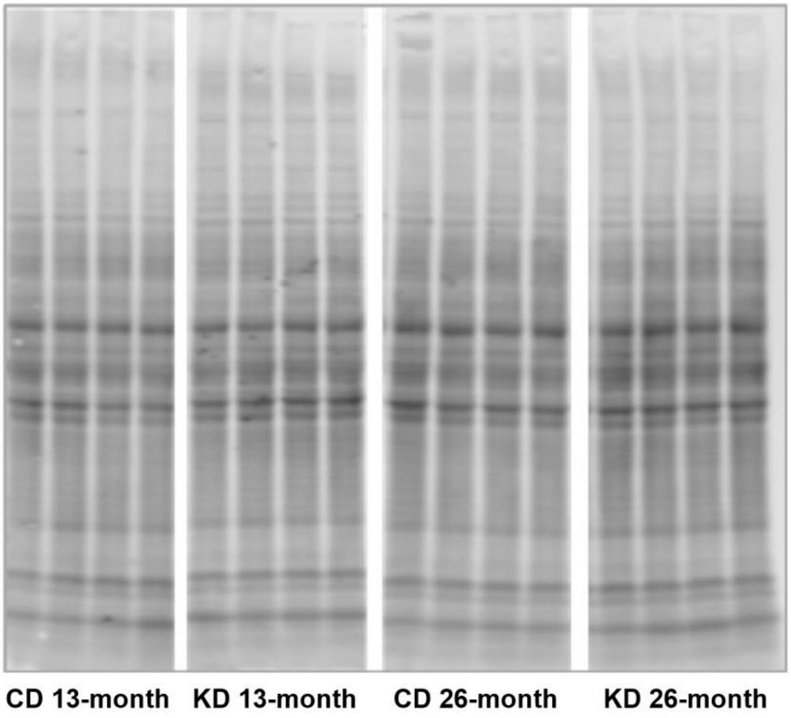

Supplementary Figure 4. Total protein normalization for western blot analysis of kidney.
